# Parameter Calculation and Rotor Structure Optimization Design of Solid Rotor Induction Motors

**DOI:** 10.3390/s25092929

**Published:** 2025-05-06

**Authors:** Hao Xu, Jinghong Zhao, Sinian Yan

**Affiliations:** School of Electrical Engineering, Naval University of Engineering, Wuhan 430033, China; xuhao10141205@163.com (H.X.); zhaojinghong@163.com (J.Z.)

**Keywords:** solid rotor induction motors, generalized analytical model of magnetic fields, structural optimization, finite element simulation, prototype experiment

## Abstract

**Highlights:**

**What are the main findings?**

- A generalized electromagnetic analytical model for solid rotor motors has been developed, which can consider the effects of rotor structure, saturation and eddy currents.
- Optimized design of slotted and squirrel cage solid rotor induction motors is presented.

**What are the implications of the main findings?**

- A simple and accurate method for calculating electromagnetic parameters of solid rotor induction motors is provided.
- Technical support for the optimal design of solid rotor induction motors is provided.

**Abstract:**

Solid rotor induction motors have a solid body rotor, which leads to low efficiency and power factor, and currently, the rotor is mostly optimized by slotted and squirrel cage structures. A generalized multilayer analytical model for different rotor structures is established, which can consider the effects of rotor eddy currents and saturation, based on which a generalized equivalent circuit model is established. The effects of number of slots, depth of slots, width of slots, squirrel cage material and end ring thickness on rotor impedance, torque and rotor losses are analyzed. On this basis, with efficiency, power factor, starting torque and starting current as the optimization objectives, and the number of slots, slot depth, slot width, squirrel cage material and end ring thickness as the optimization variables, the optimization schemes of slotted rotor and squirrel cage rotor are obtained by using the three-dimensional finite element method. The theoretical analysis is verified by finite element simulation and prototype experiment, and the results show that the electromagnetic parameters of solid rotor induction motors with different rotor structures can be accurately calculated using the universal magnetic field analytical model and the universal equivalent circuit model with an error within 5.8%. Slotted and squirrel cage rotors can effectively improve the motor power factor and efficiency, but this will lead to a decrease in starting performance. For the optimization function established in this paper, compared with the smooth rotor, the performance of the squirrel cage rotor is improved by 6.08%, which verifies the accuracy and validity of this paper and the optimization design scheme.

## 1. Introduction

Solid rotor induction motors have the advantages of a good starting performance, high rotor mechanical strength, high thermal stability, high overload capacity and high reliability, and they are widely used in applications requiring frequent starting such as articulated drive platforms for robots, intelligent logistic equipment and the industrial Internet of Things [1]. However, solid rotor induction motors operate at large differential rates with a strong skin effect, small penetration depth of magnetic flux, eddy currents and magnetic flux mainly concentrated on the rotor surface (making the rotor surface area highly saturated), complex rotor electromagnetic characteristics, and difficult calculation of rotor parameters. At the same time, the rotor surface loss is large, heating is serious, and there are certain disadvantages in the force–energy indexes, such as output power, efficiency, torque, etc. [2,3,4].

In order to improve the force–energy index of the solid rotor motor, scholars have carried out lots of research on rotor structure optimization, and their main idea is to cut off the eddy current paths and improve the magnetic field distribution on the rotor surface. The main measures include rotor slotting [5,6,7], squirrel-cage solid rotor [8,9], copper cladding on the rotor surface [10] and so on. Among them, reference [5] investigated the effect of slot-size parameters on the starting torque of solid rotor motors to obtain the slotting depth and slotting width when the performance is optimal. Reference [8] investigated the effect of the slot size of squirrel cage solid rotor on motor air gap flux density harmonics and motor losses. Reference [10] used the shielding effect of the eddy current magnetic field to analyze the effect of copper cladding on the rotor surface on the harmonic components of the air gap magnetic field and the eddy current loss of the rotor, and investigated the relationship between the thickness of the copper cladding and the eddy current loss. In addition, the guide bar failure prone to squirrel cage rotor is also the focus of motor design attention; reference [11] designed a detection and isolation program which provides accurate isolation of early rotor guide bar failure.

The rotor circuit of a solid rotor induction motor is integrated with the magnetic circuit, constituting a closed loop of induced current and main flux at the same time, resulting in an irregular distribution of the magnetic field of the solid rotor; thus, the rotor parameters are distributed and strongly correlated with the slew rate and magnetic field strength, which makes it impossible to compute the impedance of the solid rotor using Ohm’s law [12]. Optimization of the rotor structure further increases the number of electromagnetic field solution domains and diversifies the boundary conditions, resulting in more complex magnetic field distribution and parameter calculation of the motor. The current electromagnetic field analysis methods for solid rotor motors mainly include the penetration depth method, the magnetic level analysis method, the multilayer theory method, the angle-preserving transformation method and the finite element method [13]. Reference [14] calculated the magnetic field distribution and equivalent circuit parameters of a solid rotor induction motor considering the influence of the stator cogging effect using the magnetic potential resolution method. Reference [15] utilized the sub-domain method to establish an analytical model of the magnetic field of a solid rotor induction motor, which is capable of accurately solving the inductance of the motor under different operating conditions. Reference [16] calculated the rotor impedance, torque and efficiency of a double stator solid rotor axial flux induction motor using the multilayer theory method. Reference [17] developed an equivalent circuit model of a new solid rotor induction motor with toroidal windings using the multilayer theory method, which is capable of accurately calculating the steady-state performance of the motor. Reference [18] analyzed the electromagnetic characteristics of a solid rotor motor using the conformal transformation method, which can consider the end effects of the solid rotor. Reference [19] analyzed the impedance parameters and torque characteristics of a solid rotor motor using the finite element method. In summary, the current solid rotor motors with different rotor structures mostly use different methods to calculate and optimize the electromagnetic parameters of the motors, and there is a lack of universal magnetic field analysis model and equivalent circuit model, which can comprehensively consider the effects of the solid rotor saturation effect, rotor material and rotor structure, and other factors. The three-dimensional finite element method can accurately calculate the motor parameters, but the calculation time is too long, and the mathematical relationship between the structural dimensions and electromagnetic parameters cannot be obtained [20].

In this paper, the multilayer analytical method and equivalent magnetic circuit method are used to establish a general magnetic field analytical model for solid rotor induction motors applicable to different rotor structures. On this basis, based on the surface impedance theory, a general equivalent circuit model is established in the form of a cascade, and the rotor impedance, rotor loss and torque are obtained by calculating the rotor impedance. The effects of the performance of the motors in terms of the number of slots, depth of the slots, width of the slots, the squirrel cage material and the thickness of the end rings are analyzed. Then, taking the slotted solid rotor and squirrel cage solid rotor as an example, the optimization scheme is obtained by taking efficiency, power factor, starting current and starting torque as the optimization objectives, and using the transient finite element method to optimize the motor in terms of the number of slots, slotting depth, slotting width, squirrel cage material and end ring thickness. Finally, the magnetic field analytical model and structure optimization method are verified using the finite element simulation and prototype test, and the enhancement of motor performance by slotting and squirrel cage structure is analyzed.

The main innovations of this paper are as follows:

(1) A multilayer electromagnetic analytical model considering the saturation effect of solid rotor has been established by combining the equivalent magnetic circuit method, the layered method and the magnetization curve, which is capable of accurately solving the magnetic field distribution and electromagnetic parameters of solid rotor induction motors with different rotor structures.
(2) A multi-objective optimization design of solid rotor induction motors with different rotor structures has been carried out using analytical and finite element methods, and this method improves the design efficiency of the motors.

## 2. Electromagnetic Field Modeling

### 2.1. Motor Structure

**Figure 1:**
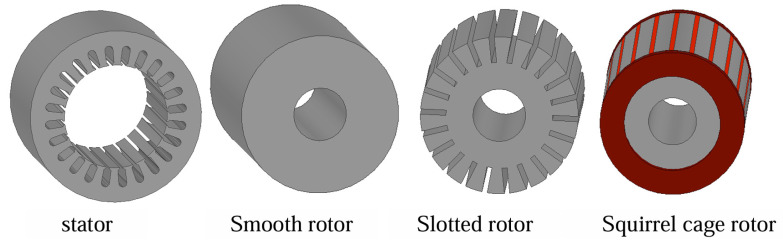
Structure of solid rotor induction motors.

As shown in Figure 1, a solid rotor induction motor consists of a stator core, windings and a solid rotor. The structural parameters of a smooth solid rotor induction motor are shown in Table 1.

**Figure 2:**
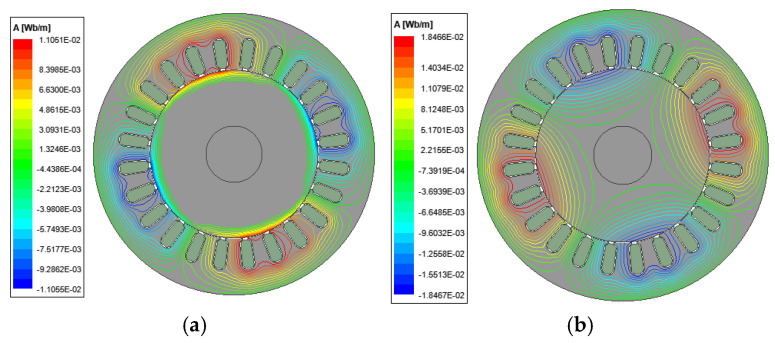
Distribution of magnetic lines of force for smooth solid rotor induction motors at different slips. (**a**) s = 0.9. (**b**) s = 0.05.

Figure 2 illustrates the distribution of magnetic lines of force in a smooth solid rotor induction motor at different slips. The results show that the magnetic lines of force do not follow an exact path within the solid rotor due to the fact that the magnetic and electric circuits of the solid rotor are merged into one, and the magnetic lines of force are gathered on the surface of the rotor at the large slip. With the reduction in the slip, the penetration depth of the magnetic lines of force increases. The distribution of the magnetic lines of force is similar to that of an ordinary squirrel-cage rotor induction motor at the rated slip.

### 2.2. Generalized Multilayer Magnetic Field Analytical Model

**Figure 3:**
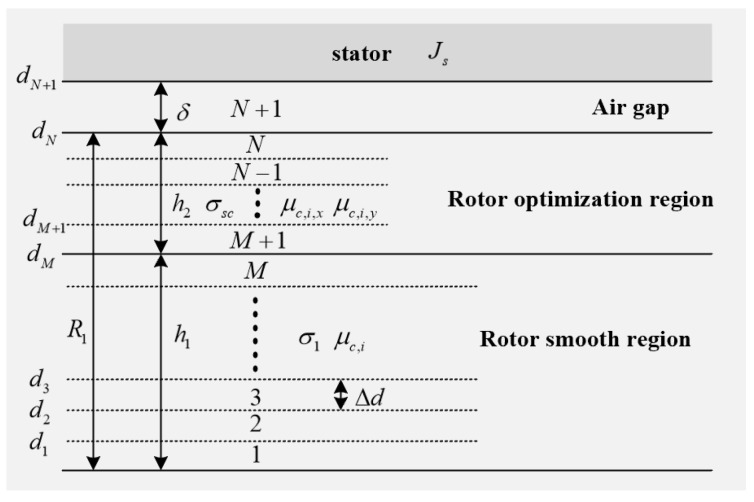
Generalized multilayer magnetic field analytical model.

To analyze the magnetic field distribution of solid rotor induction motors with different rotor structures, the general magnetic field analysis model, shown in Figure 3, is established, and the equivalent magnetic circuit method is used to equate the slotted rotor and squirrel-cage rotor to a smooth anisotropic solid rotor. The general magnetic field analysis model takes the solid rotor as the reference system, with the rotor axis as the coordinate origin; the *x*-axis direction corresponds to the tangential direction of the motor, the *y*-axis direction corresponds to the radial direction of the motor and the *z*-axis direction corresponds to the axial direction of the motor. The model can be divided into *N* layers of equal height, where the first layer of the rotor is close to the axis and layer *N* is adjacent to the air gap. The smooth region of the solid rotor is divided into *M* layers of equal height, and the optimized region for solid rotors is divided into *N-M* layers of equal height. The permeability of each layer of the rotor is constant and unequal. Since the permeability of the air gap is known, it is divided into the *N* + 1 layer.

In Figure 3, the anisotropy of ferromagnetic materials can be neglected in the rotor smooth region, assuming that the permeability of each layer is constant and not equal to each other. $\mu_{i}$ is the relative magnetic permeability of layer *i* in the smooth region of the rotor and $\sigma_{1}$ is the electrical conductivity in the smooth region of the rotor. By contrast, the rotor optimization region has a variety of material properties and complex boundary conditions with non-negligible anisotropy. $\mu_{i,x}$ is the relative permeability in the x-direction of layer *i* of the rotor optimization region, $\mu_{i,y}$ is the relative permeability in the y-direction of layer *i* of the rotor optimization region and $\sigma_{sc}$ is the equivalent conductivity of the rotor optimization region. $d_{i}=i\Delta d$ is the distance from the upper surface of each layer to the axis of the rotor, $\Delta d=R_{1}/N$ is the height of each layer of the rotor, δ is the length of the air-gap and $J_{s}$ is the equivalent current layer density of the stator which can be expressed as

(1) $$\;J_{s}=\frac{\sqrt{2}mN_{1}k_{w1}I_{m}}{kp\tau }e^{j(\omega_{s}t-a_{k}x_{s})},$$

where m is the number of phases, $N_{1}$ is the number of series turns per phase, $k_{w1}$ is the winding coefficient, $I_{m}$ is the rms value of the stator current, p is the number of pole pairs, τ is the pole pitch, k is the number of spatial harmonics and $\omega_{s}$ is the angular frequency of the stator current, $a_{k}=k\pi /\tau$.

Since the magnetic field distribution inside the solid rotor has a close relationship with the slip, it is analyzed under the rotor reference system in this paper. The stator–rotor reference system transformation relationship is as follows: $\omega_{s}t-a_{k}x_{s}=s\omega_{s}t-a_{k}x_{r}$, $y_{s}=y_{r}$,$z_{s}=z_{r}$.

The following assumptions are made for the purpose of analysis:

(1) End coefficients are used to consider the effect of solid rotor end effects.
(2) infinite magnetic permeability of the stator core.
(3) The influence of hysteresis effect of the core is neglected.
(4) The value of the radial magnetic field remains constant through the air gap.

Based on Maxwell’s equations, a generalized vector magnetic potential expression can be expressed as follows:

(2) $$\left\{\begin{array}{c} \frac{\partial {A_{i}}^{2}}{\partial x^{2}}+\frac{\partial {A_{i}}^{2}}{\partial y^{2}}=js\omega_{s}\mu_{i}\mu_{0}\sigma_{1}A_{i},i\in [1,M]\\ \frac{1}{\mu_{i,y}}\frac{\partial {A_{i}}^{2}}{\partial x^{2}}+\frac{1}{\mu_{i,x}}\frac{\partial {A_{i}}^{2}}{\partial y^{2}}=js\omega_{s}\mu_{0}\sigma_{sc}A_{i},i\in [M+1,N]\\ \;\frac{\partial {A_{N+1}}^{2}}{\partial x^{2}}+\frac{\partial {A_{N+1}}^{2}}{\partial y^{2}}=0 \end{array}\right.,$$

The solution can be obtained as follows:

(3) $$\left\{\begin{array}{c} \begin{array}{l} A_{i}=\sum_{b=1}^{\infty }E_{b}^{i}\left\{\begin{array}{l} \sinh\xi_{b}^{i}(y-d_{i-1})+\\ F_{b}^{i}\cosh\xi_{b}^{i}(y-d_{i-1}) \end{array}\right\}e^{j(s\omega_{s}t-a_{k}x_{r})},i\in [1,M]\\ A_{i}=\sum_{e=1}^{\infty }E_{e}^{i}\left\{\begin{array}{l} \sinh\xi_{e}^{i}(y-d_{i-1})+\\ F_{e}^{i}\cosh\xi_{e}^{i}(y-d_{i-1}) \end{array}\right\}e^{j(s\omega_{s}t-a_{k}x_{r})},i\in [M+1,N] \end{array}\\ A_{N_{3}+1}=\sum_{v=1}^{\infty }E_{v}^{N+1}\left\{\begin{array}{l} \sinh a_{v}(y-d_{N_{3}})+\\ F_{v}^{N+1}\cosh a_{v}(y-d_{N_{3}}) \end{array}\right\}e^{j(s\omega_{s}t-a_{k}x_{r})} \end{array}\right.,$$

where $E_{b}^{i}$, $F_{b}^{i}$, $E_{e}^{i}$, $F_{e}^{i}$, $E_{v}^{N+1}$ and $F_{v}^{N+1}$ are coefficients to be determined, b, e and v are the magnetic field harmonic orders in the rotor smooth region, rotor-optimized region and air-gap region, respectively, av=vπτ, $\xi_{b}^{i}$ and $\xi_{e}^{i}$ can be expressed as follows:

(4)    ξbi=(bπτ)2+jsωsμ0μiσ1ξei=(eπτ)2μi,xμi,y+jsωsμ0μi,xσsc,

The boundary conditions are shown as follows:

(5) $$\;\;\;\;\;\;\left\{\begin{array}{c} \;{\left.A_{i}\right|}_{y=d_{i}}={\left.A_{i+1}\right|}_{y=d_{i}}\\ {\left.\frac{1}{\mu_{i,x}}\frac{\partial A_{i}}{\partial y}\right|}_{y=d_{i}}={\left.\frac{1}{\mu_{i+1,x}}\frac{\partial A_{i+1}}{\partial y}\right|}_{y=d_{i}}\\ \;{\left.\frac{1}{\mu_{0}}\frac{\partial A_{N+1}}{\partial y}\right|}_{y=d_{N+1}}=J_{s}\\ {\left.A_{1}\right|}_{y=0}=0 \end{array}\right.,$$

Substituting the boundary condition Equation (5) into the vector magnetic potential solution Equation (3), the relationship between the coefficients can be obtained, and the coupling relationship between the air gap and the rotor surface is processed by using the Fourier series method. Then, the expressions of vector magnetic potentials can be obtained for the air gap and each layer of the solid rotor.

The equivalent reluctance in the optimized region of the rotor is analyzed by taking the slotted solid rotor and squirrel cage solid rotor induction motor as an example.

**Figure 4:**
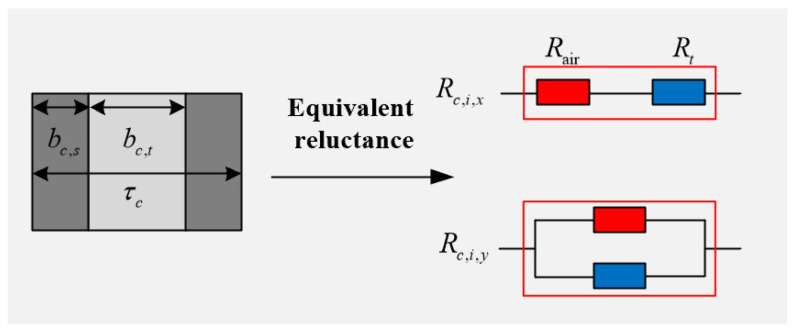
Schematic diagram of equivalent reluctance of the slotted solid rotor.

The equivalent magnetoresistance of the slotted solid rotor is shown in Figure 4, where the magnetoresistance in the *x*-direction of the slotted region is equivalent to the series connection of the tooth reluctance and the air reluctance, the magnetoresistance in the *y*-direction is equivalent to the parallel connection of the tooth reluctance and the air reluctance and the electrical resistance is equivalent to the tooth resistance equally distributed in the slotted region.

In Figure 4, $b_{c,s}$ is the slotted width, $b_{c,t}$ is the tooth width, $\tau_{c}$ is the tooth pitch of the slotted rotor, $R_{\mathrm{air}}$ is the air reluctance, $R_{t}$ is the tooth reluctance, $R_{c,i,x}$ is the equivalent reluctance in the *x*-direction of layer *i* of the slotted rotor and $R_{c,i,y}$ is the equivalent reluctance in the *y*-direction of layer *i* of the slotted rotor. At this point, the equivalent magnetic permeability and equivalent electric permeability of the rotor optimization region of the slotted solid rotor induction motor can be expressed as

(6) $$\;\;\;\;\left\{\begin{array}{c} \mu_{c,i,x}=\frac{\mu_{c,i}\tau_{c}}{b_{c,t}+b_{c,s}\mu_{i}}\\ \;\;\mu_{c,i,y}=\mu_{c,i}\frac{b_{c,t}}{\tau_{c}}+\frac{b_{c,s}}{\tau_{c}}\\ \;\sigma_{2}=\sigma_{1}\frac{b_{c,t}}{\tau_{c}} \end{array}\right.,$$

where $\mu_{c,i}$ is the relative permeability of the teeth in the slotted region of the rotor, $\mu_{c,i,x}$ is the relative permeability of the *i*th layer of the slotted rotor in the *y*-direction, $\;\mu_{c,i,y}$ is the relative permeability in the *y*-direction of the *i* layer of the slotted rotor and $\sigma_{2}$ is the equivalent conductivity of the slotted rotor.

**Figure 5:**
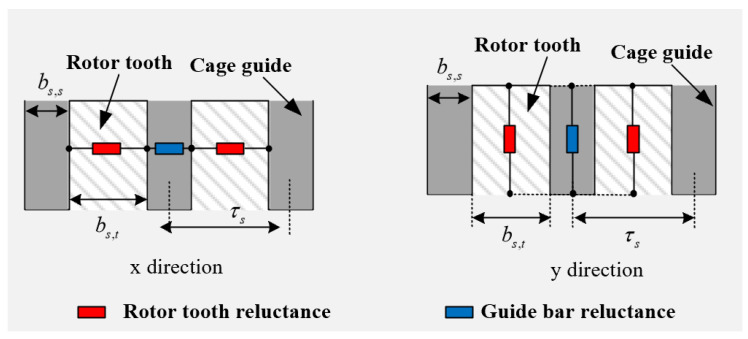
Schematic diagram of the equivalent reluctance of the squirrel cage solid rotor.

The equivalent reluctance of the squirrel cage solid rotor is shown in Figure 5, where the squirrel cage region can be approximated as an anisotropic homogeneous material, considering the reluctance in the *x*-direction to be the series connection of the tooth reluctance and the guide bar reluctance, and the reluctance in the *y*-direction to be equivalent to the parallel connection of the tooth reluctance and the air reluctance.

In Figure 5, $\tau_{s}$ is the tooth pitch of the squirrel cage rotor, $b_{s,t}$ is the tooth width and $b_{s,s}$ is the width of the guide strip. The equivalent magnetic permeability and equivalent electrical conductivity of the optimized region of the rotor of the squirrel cage solid rotor induction motor are obtained from the equivalent magnetic circuit method:

(7) $$\;\;\;\;\;\left\{\begin{array}{c} \mu_{s,i,x}=\frac{\mu_{s,i}\tau_{s}}{b_{s,t}+b_{s,s}\mu_{s,i}}\\ \;\;\mu_{s,i,y}=\mu_{s,i}\frac{b_{s,t}}{\tau_{s}}+\frac{b_{s,s}}{\tau_{s}}\\ \;\sigma_{3}=\frac{\sigma_{1}b_{s,t}+\sigma_{cage}b_{s,t}}{\tau_{s}} \end{array}\right.,$$

where $\mu_{s,i}$ is the relative permeability of the rotor material in layer *i* of the squirrel cage rotor in layer *i* of the rotor material, $\mu_{s,i,x}$ is the relative permeability in the *x*-direction of layer *i* of the squirrel-cage rotor, $\mu_{s,i,y}$ is the relative permeability in the *y*-direction of layer *i* of the squirrel-cage rotor, $\sigma_{3}$ is the equivalent conductivity of the squirrel-cage rotor and $\sigma_{cage}$ is the conductor conductivity.

### 2.3. Generalized Equivalent Circuit Model

Based on the multilayer analytical general model of magnetic field, the results of electromagnetic field analysis are presented in the form of surface impedance so as to obtain an equivalent circuit in the form of cascade, which is able to clearly represent the relationship between factors such as rotor geometry, material properties and rotor impedance. The expressions for the electric field strength and magnetic field strength of each layer of the solid rotor are shown as follows:

(8) $$\;\;\;\left\{\begin{array}{c} E_{z,i}={\left.-\frac{\partial A_{i}}{\partial t}\right|}_{y=d_{i}}\\ H_{x,i}={\left.\frac{1}{\mu_{0}}\frac{\partial A_{i}}{\partial y}\right|}_{y=d_{i}} \end{array}\right.$$

From the surface impedance theory, the relationship between the surface impedance of layer *i* and the surface impedance of layer *i* is given by

(9) $$\;\;Z_{i}=\frac{E_{z,i}}{H_{x,i}}=Z_{\sigma i}+\frac{1}{\frac{1}{Z_{\mu i}}+\frac{1}{Z_{\sigma i}+Z_{i-1}}},$$

where $E_{z,i}$ is the electric field strength in the z-direction at layer *i*, $H_{x,i}$ is the magnetic field strength in the *x*-direction at layer *i*, $Z_{\sigma i}$ is the series impedance at layer *i* and $Z_{\mu i}$ is the tapped impedance at layer *i*. $Z_{\sigma i}$ and $Z_{\mu i}$ are denoted as

(10) $$\;\;Z_{\sigma i}=\overset{-}{Z_{i}}\tanh(\xi_{i}\Delta d/2)\approx \overset{-}{Z_{i}}\xi_{i}\Delta d/2,$$

(11) $$\;\;Z_{\mu i}=\overset{-}{Z_{i}}/\sinh(\xi_{i}\Delta d)\approx \overset{-}{Z_{i}}/(\xi_{i}\Delta d),$$

where $\overset{-}{Z_{i}}$ is the characteristic wave impedance of layer *i*, which can be expressed as

(12) $$\;\;\overset{-}{Z_{i}}=js\omega_{s}\mu_{i}/\xi_{i}$$

**Figure 6:**
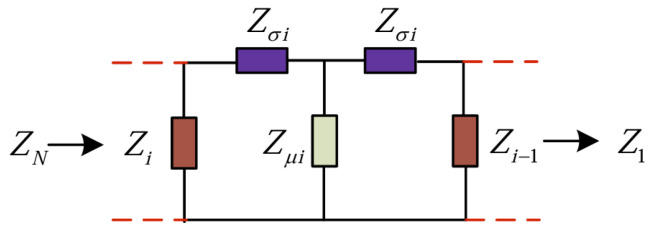
Generalized equivalent circuit model.

It can be seen that $Z_{i}$ is the cascade impedance of the layered impedances $Z_{\sigma i}$ and $Z_{\mu i}$ of this layer, and the surface impedance $Z_{i-1}$ of its sublayer. Sequentially, it can be obtained that $Z_{N}$, which represents the surface impedance of the rotor. Based on this, the equivalent circuit model in cascade form shown in Figure 6 is obtained.

The surface impedance $Z_{1}$ of the first layer of the rotor can be expressed as

(13) $$\;\;Z_{1}=\frac{E_{z,1}}{H_{x,1}}=-j\frac{ns\omega_{s}\mu_{1}}{\xi_{1}}$$

Meanwhile, $Z_{N}$ can be equated to the rotor impedance per unit axial length at each point on the circumference of the rotor surface, so the rotor impedance converted to the stator side can be expressed as

(14) $$\;\;Z_{r}=\frac{4m{\left(N_{1}k_{w1}\right)}^{2}l_{ef}}{s\pi D_{3}}Z_{N}=R_{r}+jX_{r},$$

where $l_{ef}$ is the effective length of the rotor.

The rotor current $I_{r}$ can be expressed as

(15) $$\;I_{r}=\frac{\pi D_{3}}{2\sqrt{2}mN_{1}K_{w1}}H_{x.N}$$

According to the generalized analytical model of magnetic flux, the Pointers vector into the rotor surface is

(16) $$\;\;\;S_{r}=-\frac{1}{2}{\left.E_{z,N}\right|}_{y=d_{N}}{\left.{H_{x,N}}^{*}\right|}_{y=d_{N}},$$

where ${\left.{H_{x,N}}^{*}\right|}_{y=d_{N}}$ is the conjugate value of the x-direction component of the magnetic field strength at the rotor surface.

The electromagnetic torque can be expressed as

(17)    Te=Re2p2τlefSrsωs

The rotor loss can be expressed as

(18)      Pr=2pτlefReSr

## 3. Optimized Design of Rotor Structures

### 3.1. Effect of Optimization Variables on Motor Performance

**Figure 7:**
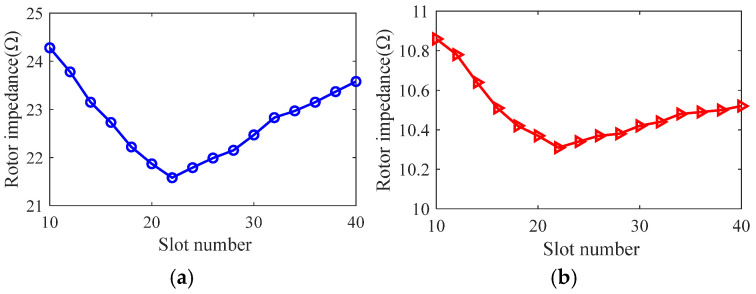
The effect of the number of slots on rotor impedance. (**a**) s = 0.1. (**b**) s = 0.5.

**Figure 8:**
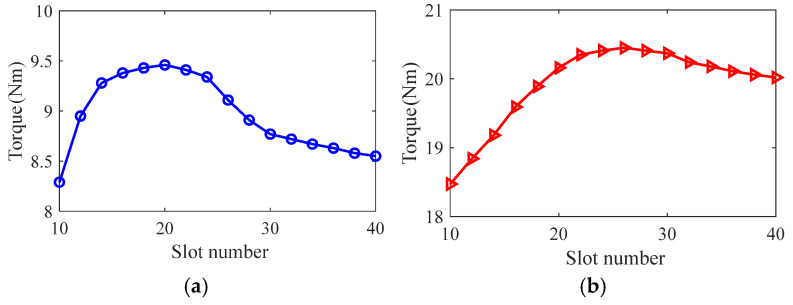
The effect of the number of slots on torque. (**a**) s = 0.1. (**b**) s = 0.5.

**Figure 9:**
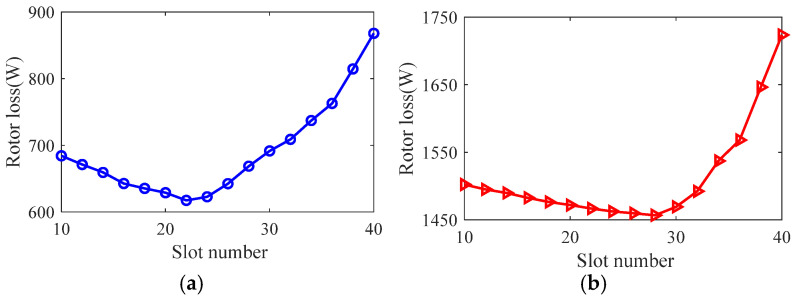
The effect of the number of slots on rotor loss. (**a**) s = 0.1. (**b**) s = 0.5.

**Figure 10:**
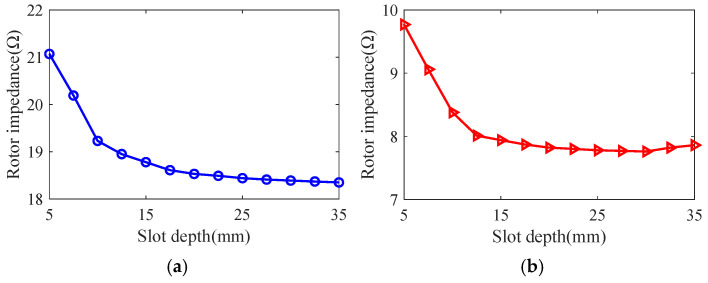
The effect of slotted depth on rotor impedance. (**a**) s = 0.1. (**b**) s = 0.5.

**Figure 11:**
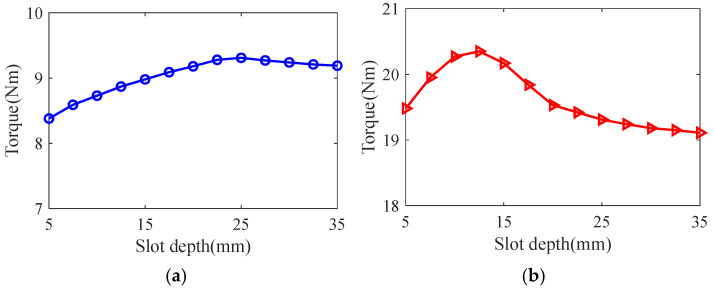
The effect of slotted depth on torque. (**a**) s = 0.1. (**b**) s = 0.5.

**Figure 12:**
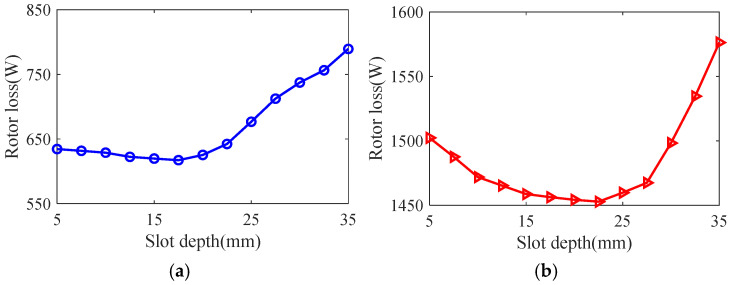
The effect of slotted depth on rotor loss. (**a**) s = 0.1. (**b**) s = 0.5.

**Figure 13:**
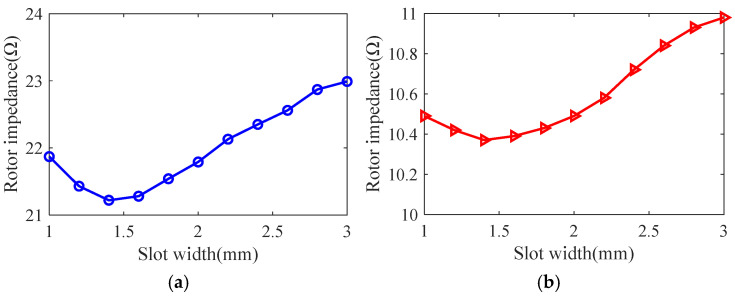
The effect of slotted width on rotor impedance. (**a**) s = 0.1. (**b**) s = 0.5.

**Figure 14:**
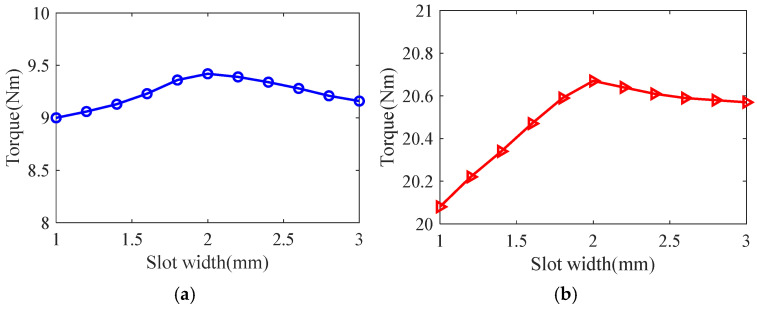
The effect of slotted width on torque. (**a**) s = 0.1. (**b**) s = 0.5.

**Figure 15:**
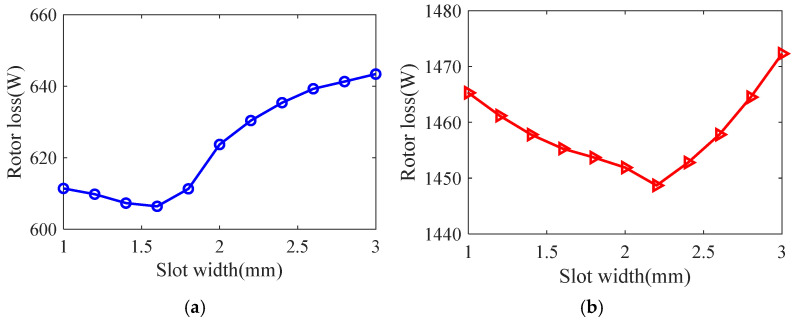
The effect of slotted width on rotor loss. (**a**) s = 0.1. (**b**) s = 0.5.

Taking the smooth solid rotor induction motor shown in Table 1 as the object of study, the effect of the number of slots, the depth of slots and the width of slots on the rotor impedance, torque and rotor losses is analyzed by the control variable method using the general magnetic field analytical model and the equivalent circuit model at the different slip, and the results are shown in Figure 7, Figure 8, Figure 9, Figure 10, Figure 11, Figure 12, Figure 13, Figure 14 and Figure 15. It can be seen that the number of slots, the depth of slots and the width of slots have a common effect on the rotor impedance, rotor loss and torque. As the number of slots, the depth of slots and the width of slots increase, the rotor impedance and rotor loss show a tendency to decrease and then increase, while the torque shows a tendency to increase. The main reason is that slotting cuts off the eddy current path on the rotor surface, leading to an increase in the effective area on the rotor surface, which reduces the rotor impedance and rotor loss, and increases the torque. However, when the number of slots, the depth of slots and the width of slots increase to a certain extent, the slot leakage flux increases and the slot leakage impedance increases, resulting in an increase in the rotor impedance, an increase in the rotor loss and a decrease in the torque.

**Figure 16:**
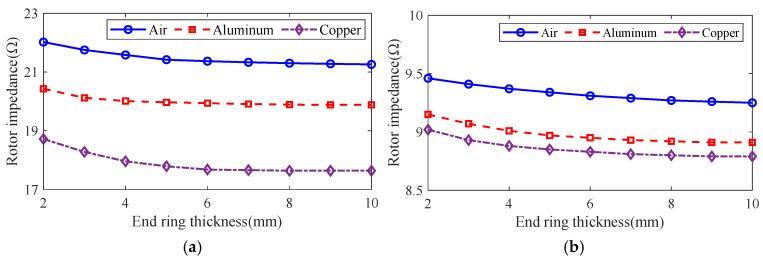
The effect of end ring thickness on rotor impedance. (**a**) s = 0.1. (**b**) s = 0.5.

**Figure 17:**
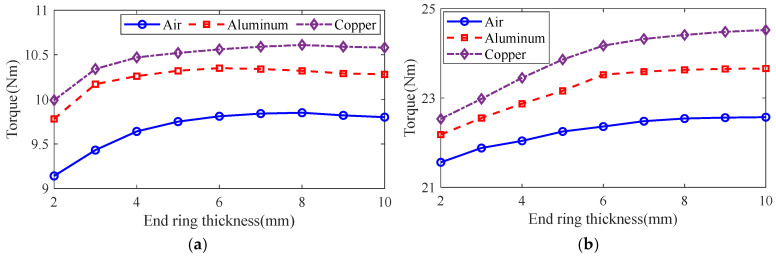
The effect of end ring thickness on torque. (**a**) s = 0.1. (**b**) s = 0.5.

**Figure 18:**
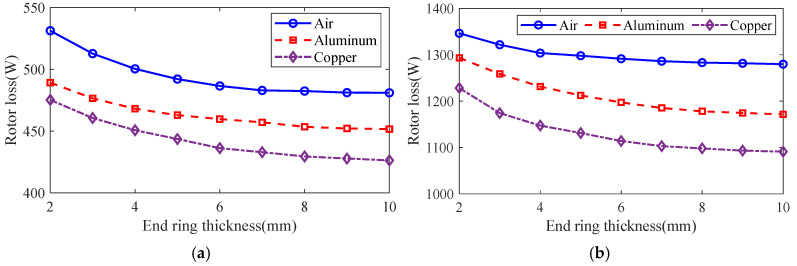
The effect of end ring thickness on rotor loss. (**a**) s = 0.1. (**b**) s = 0.5.

The control variable method is used to analyze the effect of guide strip material and end ring thickness on motor rotor impedance, torque and rotor loss at the different slip using the general magnetic field analytical model and equivalent circuit model, and the results are shown in Figure 16, Figure 17 and Figure 18. It can be seen that, with the increase in the end ring thickness, the rotor impedance and rotor loss show a trend of decreasing and then remaining basically unchanged. This is due to the increase in the end ring thickness: as the skin effect is weakened and the leakage magnetism comes to an end, the rotor impedance is reduced. As the thickness of the end ring reduces to a certain value, the skin effect and the end of the leakage flux of the effect no longer changes, and the rotor impedance remains basically unchanged. The change in rotor impedance will cause the change in end current, which makes the rotor loss also show the same trend of decreasing first and then remaining basically unchanged. At the same time, with the increase in the end ring thickness, the torque shows the trend of increasing and then slightly decreasing, which is due to the increase in the end ring thickness, so that the rotor impedance decreases and the rotor current increases. But, when the thickness of the end ring increases to a certain degree, the rotor resistance and current no longer change, and the thickness of the end ring will lead to the increase in the leakage flux, leakage reactance and other parameters, which will lead to the torque no longer increasing, or even slightly decreasing. In addition, compared with the aluminum strip, the rotor impedance and rotor loss are smaller, and the torque is larger when using copper strips. This is because the magnetic permeability of copper and aluminum is similar, while the electrical conductivity of copper is significantly larger than that of aluminum, which makes the resistance of the copper strips smaller, and produces less loss under the same current. At the same time, the copper strip can better conduct the current, while the skin effect has less influence, being better coupled with the stator magnetic field to produce greater torque.

### 3.2. Optimization Goal Analysis

Optimization objectives for solid rotor induction motors depend on motor characteristics and application requirements.

(1) Taking the product of efficiency η and power factor cosφ as the first optimization objective, it can be expressed as

(19) $$\;\;\;f_{1}(x)=\max[\eta \cos\varphi ]$$

(2) The ratio of starting torque $T_{st}^{*}$ to starting current $I_{st}^{*}$ as the second optimization objective can be expressed as

(20) $$\;\;\;f_{2}(x)=\max[\frac{T_{st}^{*}}{I_{st}^{*}}]$$

In summary, the expression of the integrated multi-objective function constructed can be expressed as

(21) $$\;\;obj_{SRIM}=\max\left[\eta \cos\varphi \frac{T_{st}^{*}}{I_{st}^{*}}\right]$$

### 3.3. Optimized Design of Slotted Solid Rotors

According to the principle of induction motor stator–rotor slot fit, the number of stator slots $Q_{s}$ and the number of rotor slots $Q_{r}$ should satisfy the following condition principles.

(1) To minimize the vibration of the motor, low-order electromagnetic force wave should be avoided. And the tooth harmonics related to the number of slots in the stator and rotor are the main components of the electromagnetic force wave, and the order $\gamma_{l}$ can be expressed as
  (22) $$\;\;\gamma_{l}=\left\{\begin{array}{c} l_{2}Q_{r}+l_{1}Q_{s}\pm 2p,l_{1}l_{2}<0\\ l_{2}Q_{r}-l_{1}Q_{s},l_{1}l_{2}>0 \end{array}\right.,$$
  where $l_{i}=\pm 1,\pm 2,\pm 3\ldots ,i=1,2$.
(2) To minimize the additional losses, a close groove fit with fewer grooves should be used.
(3) To limit the asynchronous additional torque generated by the tooth harmonic magnetic potential, the following equation should be satisfied.
  (23) $$\;\;Q_{r}\leq 1.25(Q_{s}+p),$$
(4) To avoid synchronizing additional torque during motor operation, the following equation should be avoided.
  (24) $$\;\left\{\begin{array}{c} Q_{r}=Q_{s}+2p\\ Q_{r}=6pQ_{s}+2p\\ Q_{r}=Q_{s}+p \end{array}\right.,$$

**Figure 19:**
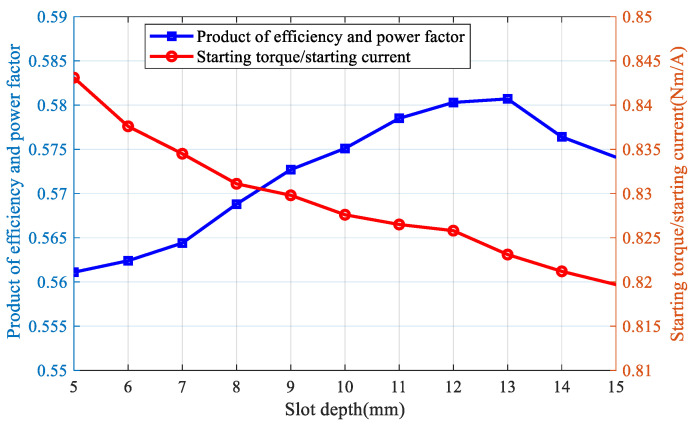
Calculation results of motor optimization index under different slot depths.

Combined with the stator side parameters, the number of rotor slots selected should be 22. On this basis, the motor performance is calculated using a three-dimensional finite-element transient field, and the depth of the rotor slots and the width of the slots are determined. The performance index $f_{1}$ and performance index $f_{2}$ are calculated for different slotting depths, and the results are shown in Figure 19. It can be seen that, with the increase in slotted depth, $f_{1}$ first increases and then decreases. This is due to the increase in slotted depth, to a certain extent, which weakened the influence of the skin effect so that the magnetic flux penetration depth increases, reducing the rotor eddy current loss and improving the power factor. However, when the depth of the slotted depth increases to a certain value, the leakage impedance increases, so that the efficiency and power factor are reduced. At the same time, with the increase in the slotting depth, $f_{2}$ shows a trend of decreasing all the time. The main reason is that, as the slotting depth increases, the rotor impedance decreases and the power factor increases, which makes the starting torque and starting current increase. However, the magnitude of the increase in the starting torque is smaller than the magnitude of the increase in the starting current, resulting in the decrease of $f_{2}$. When the slotting depth is increased to a certain point, the slot leakage impedance increases, causing the torque to decrease and the current to increase. Therefore, $f_{2}$ keeps decreasing as the slotting depth increases. In addition, from the calculation results of $f_{1}$ and $f_{2}$, the change in multi-objective function $obj_{SRIM}$ can be obtained under different slotting depths, and the results show that, when the slotting depth is taken as 12 mm, $obj_{SRIM}$ is the maximum.

The rotor impedance, torque and rotor losses show a tendency to increase and then decrease with the increase in the slotting width at the small slip. Taking the number of rotor slots as 22 and the depth of slots as 12 mm, the width of slots deepened from 0.5 mm to 3 mm with a spacing of 0.5 mm. Taking the slip as 0.05, the indexes $f_{1}$ and $f_{2}$ of the motor are calculated and the results are shown in Table 2. It can be seen that, with the increase in slotted width, $f_{1}$ increases and then decreases, $f_{2}$ gradually decreases, and the multi-objective function $obj_{SRIM}$ shows the trend of increasing and then decreasing, and reaches the maximum at 1.5 mm. In addition to this, the change of $obj_{SRIM}$ is small, in the range of 0.5 mm to 2 mm for the slotted width. $obj_{SRIM}$ decreases rapidly after the slotted width exceeds 2 mm. This is due to the fact that an appropriate increase in slotting width can improve the air gap magnetic field distribution and reduce the degree of magnetic field distortion, but when the slotting width is too large, the slot leakage reactance increases, resulting in a reduction in the motor starting performance and steady-state performance.

The smooth solid rotor induction motor shown in Table 1 is designated as the M2 prototype. According to the calculation results of the finite element transient field, this paper selects the number of rotor slots as 22, the depth of slots as 12 mm, and the width of slots as 1.5 mm as the optimization scheme of the slotted solid rotor, and names it M3 prototype, whose stator side parameters are the same as those of the M2 prototype.

### 3.4. Optimized Design of Solid Rotor with Squirrel Cage

**Figure 20:**
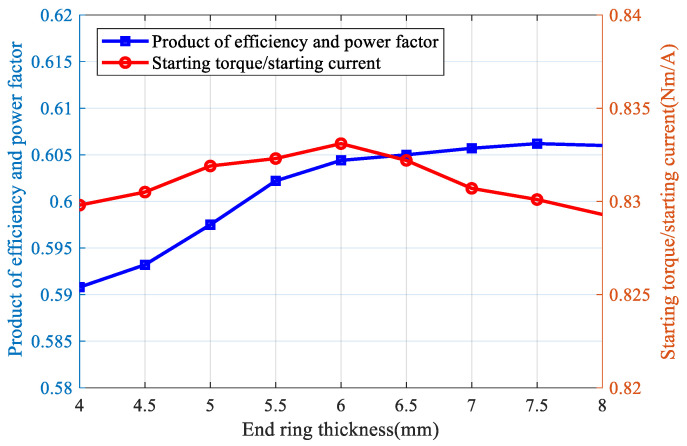
Calculation results of motor optimization index under different end ring thickness.

In order to determine the optimum end ring thickness, a three-dimensional finite element transient field is used to calculate the performance of squirrel cage solid rotor induction motor, copper bar is selected for the squirrel cage conductor, and the value of the end ring thickness ranges from 4 mm to 8 mm, with 0.5 mm as the spacing point, and when the rate of turnover is 0.05, the results of the calculations of $f_{1}$ and $f_{2}$ are shown in Figure 20. It can be seen that, with the increase in the end ring thickness, the trend of increasing $f_{1}$ slows down, while $f_{2}$ shows a trend of increasing and then decreasing, taking into account the optimization index $f_{1}$ and $f_{2}$, when the end ring thickness is 6 mm and the multi-objective function $obj_{SRIM}$ is the largest.

According to the calculation results of the finite element transient field, this paper selects the material of the guide bar as copper and the thickness of the end ring as 6 mm as the optimization scheme of the squirrel cage solid rotor, and names it as M4 prototype, whose stator side parameters are the same as those of M2 prototype, and the rotor slotting scheme is the same as that of M3 prototype.

## 4. Simulation and Experimental Verification

### 4.1. Finite Element Simulation

**Figure 21:**
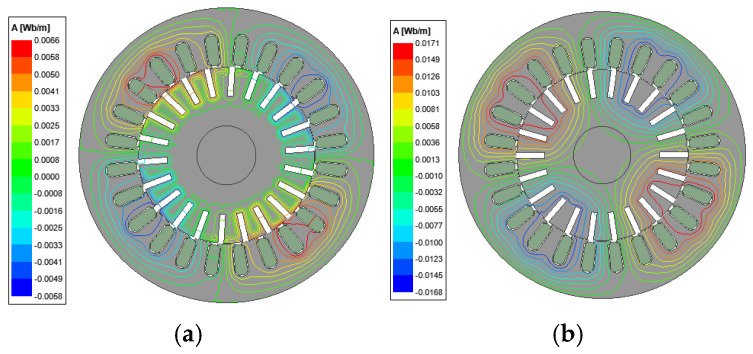
Distribution of magnetic lines of force for slotted solid rotor induction motors at the different slips. (**a**) s = 0.9. (**b**) s = 0.05.

**Figure 22:**
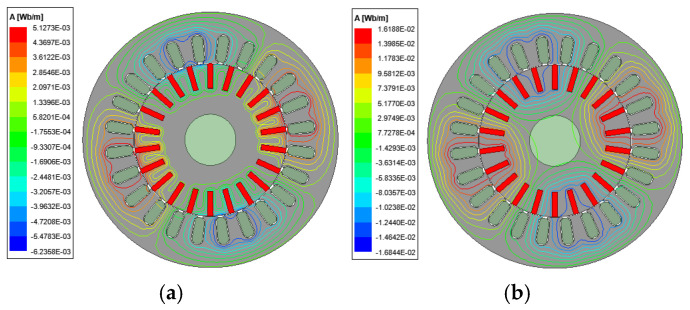
Distribution of magnetic lines of force in squirrel cage solid rotor induction motors at different slips. (**a**) s = 0.9. (**b**) s = 0.05.

According to the smooth solid rotor induction motor parameters and rotor structure optimization scheme shown in Table 1, finite element models of slotted solid rotor induction motor and squirrel cage solid rotor induction motor are constructed, and the motor air gap magnetic field, rotor impedance and rotor loss are counted to verify the correctness of the analytical calculation model. The results of the analytical calculations are obtained from the multilayer magnetic field analytical model and corrected by the end coefficients described in reference [21]. The distribution of magnetic lines for the M3 prototype and M4 prototype at different turndown rates are shown in Figure 21 and Figure 22, respectively. The results show that the rotor slots can effectively cut off the eddy current path on the rotor surface, increase the penetration depth of the magnetic flux, and increase the equivalent surface area of the rotor. The squirrel cage rotor is embedded with squirrel cage guide bars, whose conductivity is much larger than that of the solid rotor and can limit the path of the eddy currents. Both of these can effectively improve rotor magnetic field distribution.

**Figure 23:**
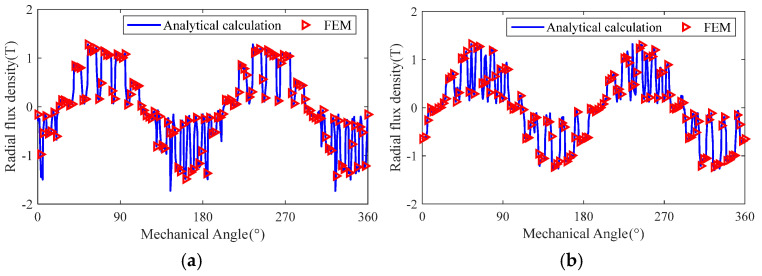
Comparison of analytical calculation results and finite element simulation results of radial air gap magnetic density motors at the different slip. (**a**) Slotted solid rotors. (**b**) Squirrel cage solid rotor.

The comparison of the analytical calculation results and finite element simulation results of the radial magnetic induction strength in the air gap of the slotted solid rotor and squirrel-cage solid rotor induction motor under rated operating conditions is shown in Figure 23. The results show that the analytical calculation results and the finite element simulation results are in good agreement, and the maximum calculation errors of the radial magnetic induction strength of the air gap of the slotted rotor and squirrel cage rotor are 3.27% and 3.93%, respectively.

**Figure 24:**
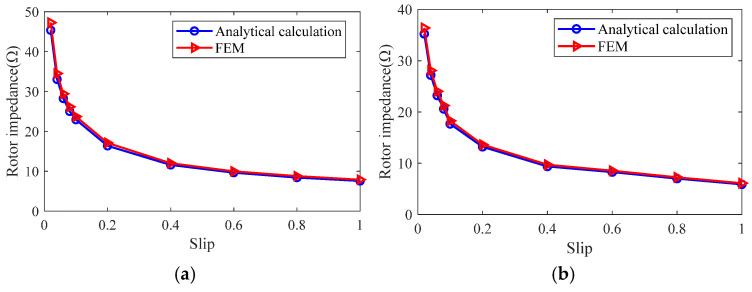
Comparison of analytical calculation results and finite element simulation results of rotor impedance. (**a**) Slotted solid rotors. (**b**) Squirrel cage solid rotor.

The rotor impedance at the different slips is calculated using analytical and finite element methods, and the results are shown in Figure 24. It can be seen that the results of the analytical calculation match the results of the finite element simulation better, and the rotor impedance calculation error of the slotted solid rotor induction motor is 2.96–4.87%, in the range of the full slip. The calculation error of rotor impedance for squirrel cage solid rotor induction motor is 3.12–5.81%.

**Figure 25:**
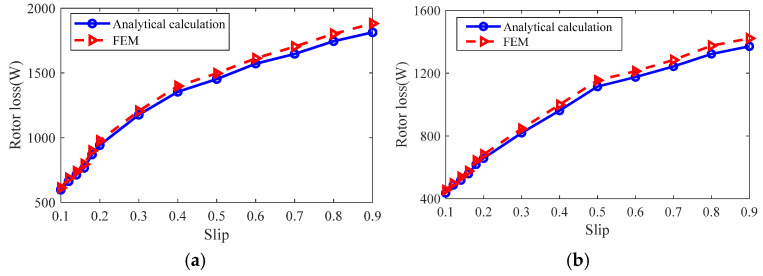
Comparison of analytical calculation results and finite element simulation results of rotor loss. (**a**) Slotted solid rotors. (**b**) Squirrel cage solid rotor.

The rotor losses at the different slip are calculated using analytical and finite element methods, and the results are shown in Figure 25. The analysis shows that the analytical calculation results and finite element simulation results are in good agreement, and the calculation error of rotor impedance of slotted solid rotor induction motor is 2.64–5.29%. The calculation error of rotor impedance of squirrel cage solid rotor induction motor is 3.46–5.67%.

From the analysis of Figure 23, Figure 24 and Figure 25, it can be seen that the magnetic field general analytical model and the equivalent circuit model can more accurately calculate the radial air-gap magnetic density, rotor impedance, and rotor loss of the slotted solid rotor and squirrel-cage solid rotor induction motors, with a maximum error of no more than 6%, which can satisfy the needs of the initial stage of the motor to quickly calculate the parameters of the motor. The main reason for the error is that the analytical calculation ignores the effect of stator core saturation.

### 4.2. Experimental Verification

**Figure 26:**
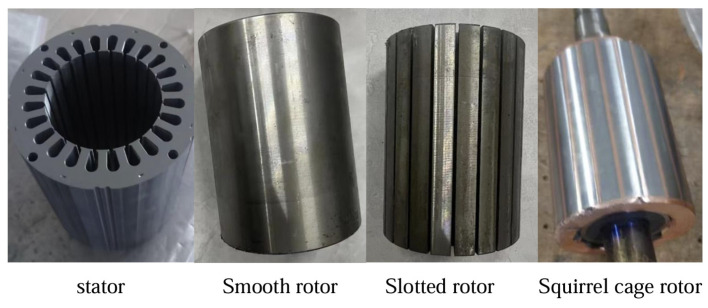
Stator and rotor structure of the motor.

A smooth solid rotor induction motor, slotted solid rotor induction motor and squirrel cage solid rotor induction motor were developed based on the parameters shown in Table 1 and the optimized design scheme. The stator–rotor structure of the motor is shown in Figure 26.

**Figure 27:**
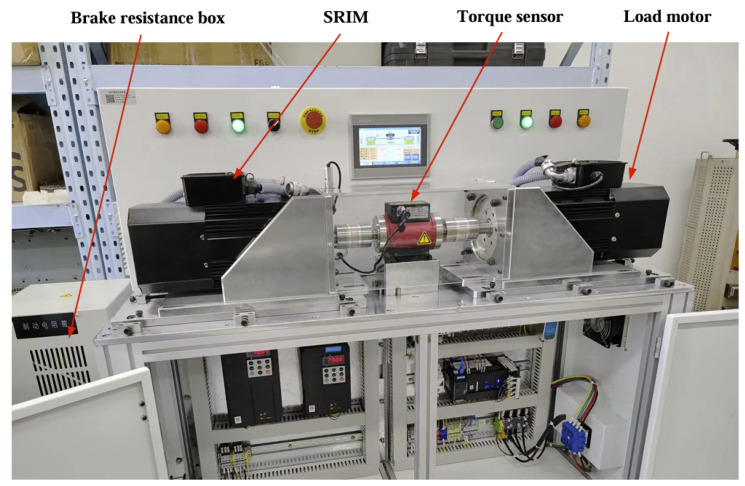
Experimental test rig for motor loads.

The motor load test rig, shown in Figure 27, was constructed. The test rig consists of a solid rotor induction motor, a human–machine interface, a torque transducer, a load motor, a braking resistor box, a programmable inverter and a three-phase power supply. The programmable inverter records the stator winding phase voltage, phase current and supply frequency parameters, from which the input power of the prototype can be calculated. Solid rotor induction motors and load motors are installed with speed sensors on the rotor shaft, which can accurately measure the speed of the motor, and torque sensors can measure the output torque of the motor, which can be calculated to obtain the output power of the motor. During the experiment, the measured waveforms of line voltage and phase current are recorded by a wave recorder because the motor windings are connected in a star shape and the neutral point is not drawn out, so the measurement of the phase voltage of the windings cannot be carried out.

**Figure 28:**
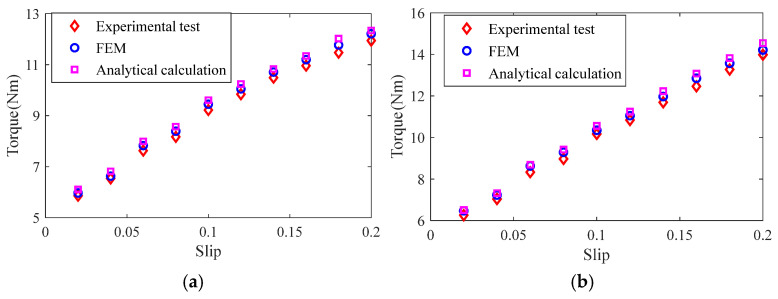
Comparison of torque of analytical calculation, finite element simulation and experimental test results. (**a**) Slotted solid rotors. (**b**) Squirrel cage solid rotor.

When the phase voltage of the stator winding is 220 V and the operating frequency is 200 Hz, the output torque of the load motor is adjusted, and load experiments are carried out on the slotted solid rotor induction motor and the squirrel cage solid rotor induction motor at different rates of rotation. The torque of the motors is measured and the experimental measurements are compared with the results of the analytical calculations and the finite element simulation, as shown in Figure 28. The results show that the consistency between the experimental measurement results and the analytical calculation results and finite element simulation results is good, in which the calculation error of the torque of the slotted solid rotor induction motor is in the range of 3.82–5.67% and that of the squirrel cage solid rotor induction motor is in the range of 4.11–5.83%. The main reason for the error is that the analytical calculation model ignores the effect of stator core saturation, and at the same time, the motor processing and test platform assembly, bearing friction generated by the additional loss and other factors, will also lead to the torque measurement value being small.

In summary, the magnetic field analytical model and equivalent circuit model established in this paper can accurately calculate the magnetic field distribution and electromagnetic parameters of solid rotor induction motors with different rotor structures. However, the model ignores the effect of temperature change on the motor material, leading to some errors in the calculation. Considering the effect of temperature change in establishing the calculation model of magnetic field and parameters will also be the focus of the next research.

**Figure 29:**
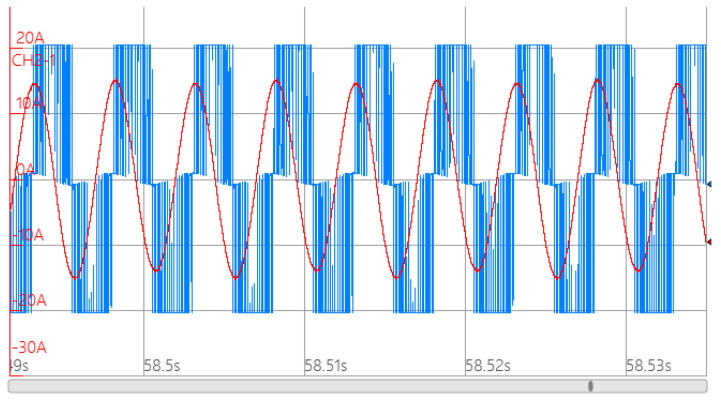
Test results of phase voltage and phase current of smooth solid rotor induction motors under rated operating conditions. The red line in the graph shows the current and the blue line shows the voltage.

Setting the effective value of the stator phase voltage to 220 V and the operating frequency to 200 Hz, the smooth solid rotor induction motor load experiment was conducted. Under rated operating conditions, the motor speed was 5700 r/min. The measurement of the line voltage and phase current waveforms are shown in Figure 29. It can be seen that the output power of the motor is 3613.8 W, the phase current is 10.4 A, the torque is 6.05 Nm and the motor efficiency is 78.7%.

Load experiments were carried out on the slotted solid rotor and squirrel cage solid rotor induction motors. The value of the stator phase voltage was 220 V, the operating frequency was 200 Hz, the value of the phase current was kept constant at 10.4. The output parameters of the M2, M3 and M4 prototypes are shown in Table 3. It can be seen that the output power, efficiency, power factor, starting torque and starting current of the M3 motor are increased by 3.87%, 3.56%, 1.98%, 7.59% and 15.42%, respectively, compared to the M2 motor, resulting in the optimization metrics $f_{1}$ being increased by 5.62%, $f_{2}$ being decreased by 6.78% and the multi-objective function $obj_{SRIM}$ being decreased by 1.55%. The output power, efficiency, power factor, starting torque and starting current of the M4 motor are increased by 9.46%, 5.97%, 5.02%, 31.25% and 37.7%, respectively, compared to the M2 motor, resulting in an increase in the optimization metrics of $f_{1}$ by 11.3%, a decrease in $f_{2}$ by 4.69% and an increase in the multi-objective function $obj_{SRIM}$ by 6.08%.

It can be seen that, in the range of small turnover rate, slotting can cut off the eddy current path on the rotor surface, improve the magnetic field distribution on the rotor surface, and weaken the influence of the skin effect, while the squirrel cage guide strip makes the rotor’s equivalent permeability increase, and the end rings weaken the influence of the end currents, so the slotting and squirrel cage structure makes the rotor impedance decrease and efficiency, power factor, and starting torque increase to a certain degree, which leads to the increase in the optimization index $f_{1}$. In addition, the reduction in rotor impedance increases the starting current, and the increase in starting current is significantly larger than the increase in starting torque, which leads to the decrease in optimization index $f_{2}$. The increase or decrease in the multi-objective function $obj_{SRIM}$ depends on the degree of change in the optimization indexes $f_{1}$ and $f_{2}$. In summary, the use of slotted and squirrel cage rotor structure can effectively improve the output power, efficiency, power factor and other force performance indicators of solid rotor induction motor, but the same will result in a reduction in starting torque per unit starting current, so the application requirements should be taken into account in selecting the structure of the solid rotor.

## 5. Conclusions

(1) A magnetic field analytical model applicable to different rotor structures is established by using the multilayer theory method and the equivalent magnetic circuit method, on the basis of which an equivalent circuit model in the form of a cascade is established, which is capable of analyzing the effects of different rotor structures and materials. Through finite element simulation and prototype test, it is proven that the magnetic field analytical model and the equivalent circuit model can accurately calculate the electromagnetic parameters of solid rotor induction motors with different rotor structures, and the calculation errors of air gap magnetic field, rotor impedance, rotor loss and torque are within 5.8%.

(2) The slotted rotor and squirrel cage rotor structures are used for the optimization of solid rotor induction motors, and the magnetic field analysis model can be used to quickly and accurately calculate the influence of the optimized parameters on the performance of the motors, and the transient finite element method is used to obtain the optimized solutions for the slotted rotor and squirrel cage rotor. Simulation and experimental results show that both the slotted rotor and squirrel cage rotor can improve the performance of solid rotor induction motors in terms of output power, efficiency and torque, but it will result in the decrease in starting performance, and it is necessary to choose the rotor structure by comprehensively considering the application requirements. For the multi-objective function constructed in this paper, the squirrel cage solid rotor induction motor performance is optimal.

## Figures and Tables

**Table 1:** Main parameters of a solid rotor induction motor.

Parametric	Notation	Value	Unit
phase	*m*	3	
rating	*P*	3.7	kW
rated frequency	*f*	200	Hz
rated speed	*n*	5700	rpm
rated voltage	*U_n_*	380	V
Number of stator slots	*Qs*	24	
polar logarithm	*p*	2	
Winding turns	*N*_1_	16	
Outer diameter of stator	*D*_1_	125	mm
Stator inner diameter	*D*_2_	75	mm
Stator inner diameter	*D*_3_	74.5	mm
Rotor inner diameter	*D*_4_	25	mm
Stator-rotor axial length	*l_ef_*	100	mm

**Table 2:** Motor performance indicators for different slotting widths.

Slotting Width/mm	$f_{1}$	$f_{2}$	$obj_{SRIM}$
0.5	0.5791	0.8279	0.4794
1	0.5801	0.8275	0.4800
1.5	0.5819	0.8265	0.4809
2	0.5803	0.8258	0.4792
2.5	0.5792	0.8214	0.4758
3	0.5787	0.8198	0.4744

**Table 3:** Experimental test results of motor performance indexes with constant phase current.

Motors	Output Power/W	Efficiency	Power Factor	$f_{1}$	Starting Torque/(Nm)	Starting Torque/A	$f_{2}$/(Nm/A)	$obj_{SRIM}$
M2	3613.8	78.7%	0.658	0.5178	22.4	25.36	0.8833	0.4574
M3	3753.6	81.5%	0.671	0.5469	24.1	29.27	0.8234	0.4503
M4	3955.7	83.4%	0.691	0.5763	29.4	34.92	0.8419	0.4852

## Data Availability

The data presented in this study are available on request from the corresponding author due to data privacy.
